# Supplementary figures and images for: CellFishing.jl: an ultrafast and scalable cell search method for single-cell RNA sequencing
Source: Genome Biol. 2019 Feb 11;20:31. doi: 10.1186/s13059-019-1639-x (PMC6371477; doi:10.1186/s13059-019-1639-x)

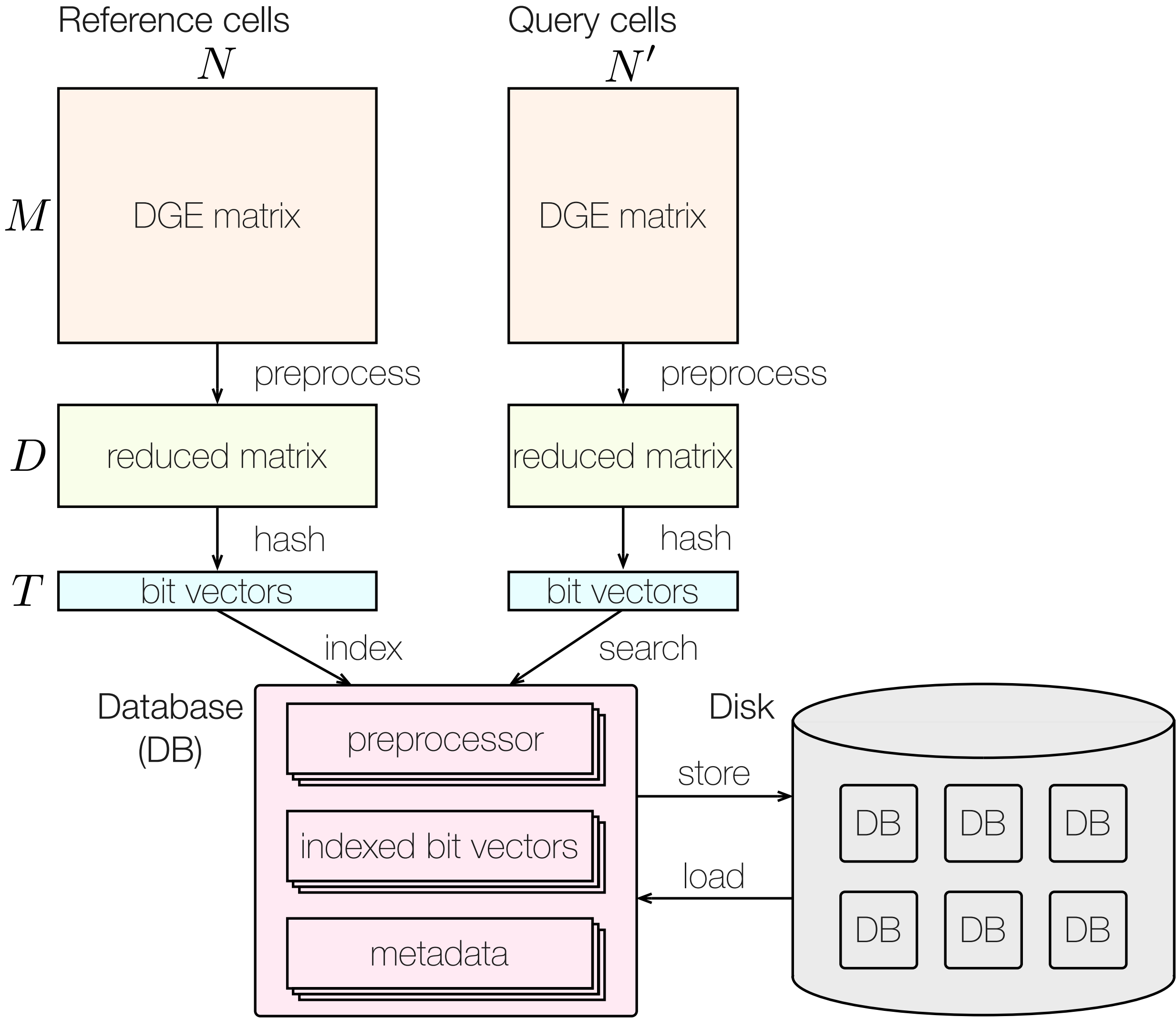

Supplement: Supplementary file 3 — Experiment scripts. Archived script files to reproduce the experiments. (TAR 81 kb) [file 13059_2019_1639_MOESM3_ESM.tar › cellfishing-experiments/figures/schematic.pdf]

subindex

filled

offsets

buckets

database

query

010111010110

110011011100

010011010101

000111011101

100011011111

010111010011

001011011000

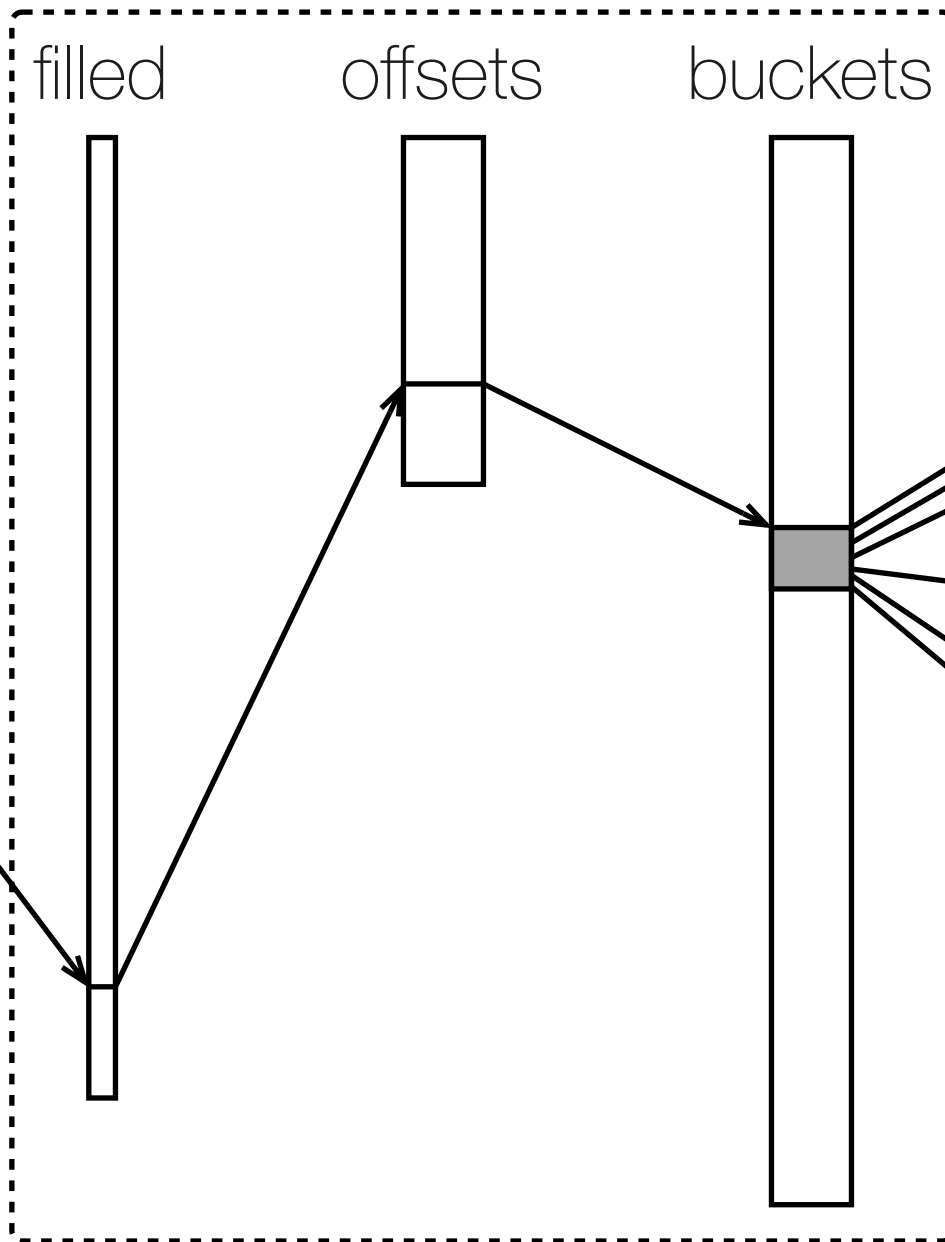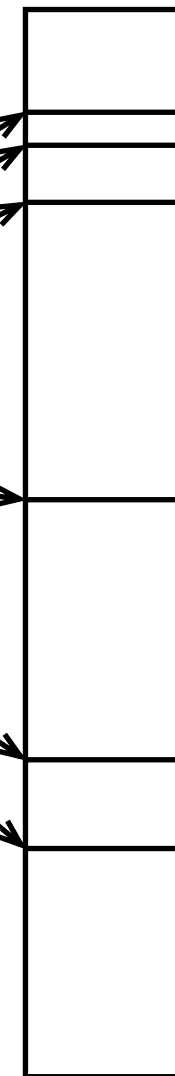

Supplement: Supplementary file 3 — Experiment scripts. Archived script files to reproduce the experiments. (TAR 81 kb) [file 13059_2019_1639_MOESM3_ESM.tar › cellfishing-experiments/figures/subindex.pdf]
