# Supplementary material for: CellFishing.jl: an ultrafast and scalable cell search method for single-cell RNA sequencing
Source: Genome Biol. 2019 Feb 11;20:31. doi: 10.1186/s13059-019-1639-x (PMC6371477; doi:10.1186/s13059-019-1639-x)
Supplement: Supplementary file 4 — Review history. (DOCX 31 kb) [file 13059_2019_1639_MOESM4_ESM.docx]

Review History

**First round of review**

**Reviewer 1**

**Are the methods appropriate to the aims of the study, are they well described, and are necessary controls included?**
I think the reported methods are according to the best practices. There is severe lack of novelty wrt the CellAtlasSearch work. I do not have any doubt about their experimental integrity.

**Are the conclusions adequately supported by the data shown?**

The claims by the authors are premised upon the experiments they conducted. They never tried to oversell.

**Are sufficient details provided to allow replication and comparison with related analyses that may have been performed?**

Yes.

**Does the work represent a significant advance over previously published studies? Please explain.**

Yes

**Is the paper of broad interest to others in the field, or of outstanding interest to a broad audience of biologists?**

Yes: Single cell data production has bumped up significantly during the past years. Fast methods for expression search are going to be really helpful.

**Comments to author:**

The authors present a method for searching similar single-cell expression profiles given a query expression vector. 

Major comments

1) I think the biggest problem with this work is its lack of novelty. It simply mirrors CellAtlasSearch for CPU users.

2) The authors used SVD, followed by hashing. It is not clear to me how it really helps in removing the batch effects. The biggest difficulty associated with single cell search is dealing with the batch effect. I request the authors to show some direct evidence (tSNE visualization, clustering etc. on data having multiple batches) shedding light on the algorithm's efficacy for batch effect removal. The statement "...at least comparable to scamp-cell …" is rather apologetic and inadequate. The authors should refer to the RCA method proposed by Li et al., as well as the canonical correlation-based method proposed by Butler et al.

3) How do the authors measure the significance of the search outcome? If I search a random expression profile, will I still retrieve matches? This must be addressed carefully and convincingly. 

4) I am not a fan of comparisons. Every landmark paper should present sufficient novel perspectives. For example, can the author show some use cases such as rare cell discovery?

Overall I think the manuscript is nicely written. The approach and experiment design are well thought out and meticulous. There is very less novelty which hinders its publication in a Tier-1 journal. However, given that it is a relatively new problem they attempted, the work will acceptable give the major concerns are addressed.

**Reviewer 2**

**Are the methods appropriate to the aims of the study, are they well described, and are necessary controls included?**
Please see my comments.

**Are the conclusions adequately supported by the data shown?**

Please see my comments.

**Are sufficient details provided to allow replication and comparison with related analyses that may have been performed?**

Please see my comments.

**Does the work represent a significant advance over previously published studies? Please explain.**

Yes

**Is the paper of broad interest to others in the field, or of outstanding interest to a broad audience of biologists?**

Yes: Cell matching in single-cell RNA-seq is an important task for biological research.

**Comments to author:**

I have multiple technique comments about this manuscript. Please see below.

Page 4: "All mouse cells (1,886 total) of the Baron2016 data set were excluded …." Do the authors mean that all human cells were excluded, since the other three datasets are all mouse data?

Page 4: "It must be noted that in our application, obtaining exact singular vectors is not particularly important;" How did the author select the number of top singular values in practice? What if the number of singular vectors needed to capture the high variability subspace corresponds to a relative high error, such as rank > 45 in Figure 2b?

Page 5: "As a result, we limited the length of the bit vectors to 128 or 256 bits in the following experiments." The example data used by the author have cell numbers on a very similar scale, so it is not a problem to use 128 or 256 bits after investigating Figure 3b. However, in real practice, such investigation is not possible. How do the authors set T in their package? Is there a way to select automatically T based on the cell number?

Page 6: In self-mapping evaluation, it would be informative to also show CellFishing's performance compared with single-cell clustering methods.

Page 9: Figure 8 is difficult to read. To better demonstrate the results on mapping across different protocols, I'd suggest the authors to also plot a heatmap containing only overlapping cell type labels from the two platforms.

Page 13: "Also, the computation of FTT is significantly faster than that of log transformation." Regardless of the computation time, how should the users select from the log or FTT transformation? How to check if the Poisson noise assumption is reasonable?

Page 14: "(3) batch effects between the query and the database are mitigated." I don't think dimension reduction can mitigate the batch effects. If there exist batch effects, some top components in the reduced space may actually represent them. 

Page 15: Is SRP-LSH proposed by the authors? There are no references for this method in the manuscript.

Page 16: In SRP-LSH, how does the dimension of the hyperplanes affect the estimation accuracy of the similarity function?

**Authors Response**

**Point-by-point responses to the reviewers’ comments:**

Original comments from reviewer #1 are written in *blue italics* and those from reviewer #2 in

*orange italics* . Our responses to the comments are in black.

Response to reviewer #1

*1) I think the biggest problem with this work is its lack of novelty. It simply mirrors*

*CellAtlasSearch for CPU users.*

This concern is understandable because both CellFishing (CF) and CellAtlasSearch (CAS) employ a similar technique (i.e., locality-sensitive hashing, LSH) to achieve the same goal of finding similar expression profiles from scRNA-seq data. In reality, it is difficult to clearly identify the differences between these two methods because the source code of CAS is not publicly available, and its implementation details are not well described in the original paper. We requested the software from the authors for evaluation in both September and October but have not yet obtained a copy. The CAS web service ( http://www.cellatlassearch.com/ ) does not accept submission of database cells from users. We would like to mention some methodological differences inferable from the paper:

1. CAS appears to cease searching at some cut-off distance from the query hash, and it was discussed that there is a tradeoff between the cut-off threshold (or the search speed) and the accuracy (see Srivastava et al., Supplementary Figure 1). This is also suggested by the following quotation from their paper: “Given a query, only a few proximal buckets are considered for an exhaustive search of nearest neighbors.” In contrast, CF can retrieve all the nearest neighbors of a query cell, as the fine technique of indexing and searching hash values (see “Indexing hash values” in the methods of our current manuscript) enables the complete retrieval of neighboring cells without exhaustive scanning of the database. This difference has importance, particularly when searching for similar but slightly different cells; CAS may stop searching before reaching such neighboring cells. We predict that our indexing technique makes CF much more rapid and accurate than other similar software.

2. Projecting the query profiles onto a low-dimensional space spanned by singular vectors computed from the variability of the database cells is crucial to reduce the number of hash bits required to reliably retrieve similar cells from the database. In addition, this projection mitigates batch effects between the query and the database, as we will clarify below. We could not find any mention of the importance of such projections in the description of CAS.

3. In the revised manuscript, we have introduced a novel function to detect query DEGs at

the single-cell level. In addition to these methodological differences, we found a statement suggesting important differences in usability in the CAS FAQ web page (visited on October 3, 2018): “A complete run on single cell dataset for a bulk query of 4 cells takes around 11-14 seconds to process. A query if 30 cells would take around 1 minutes.” This statement indicates that finding similar cells using CAS usually takes a few seconds per cell. In contrast, CF takes less than one millisecond per cell with the same scale database without GPUs (see “Scalability for large data sets” of the results). Although this may not be a fair comparison, but it is unrealistic to send tens of thousands of cells to the CAS server in order to find similar cells. In addition, our software is open-sourced and freely available under a liberal software license. Therefore, the user can easily read, modify, and extend the software for their purposes, and incorporate it as subroutine into a data processing pipeline. Requiring no GPUs also makes CF easier to use in practice.

In summary, we argue that CF has substantial differences from CAS both in methodology and usability, although both use a similar technique to summarize cellular expression profiles. We could not benchmark CAS because the software was not available during this revision time frame.

*2) The authors used SVD, followed by hashing. It is not clear to me how it really helps in removing the batch effects. The biggest difficulty associated with single cell search is dealing with the batch effect. I request the authors to show some direct evidence (tSNE visualization, clustering etc. on data having multiple batches) shedding light on the algorithm's efficacy for batch effect removal. The statement "...at least comparable to scamp-cell …" is rather apologetic and inadequate. The authors should refer to the RCA method proposed by Li et al., as well as the canonical correlation-based method proposed by Butler et al.*

We appreciate the reviewer’s comment concerning batch effects. To demonstrate the effects of query projection onto a space derived from database cells, we compared two preprocessing methods: (1) deriving variability (i.e., singular vectors corresponding to the highest singular values) from an expression matrix consisting of both query and database cells and (2) deriving variability from an expression matrix containing database cells. We visualized the two-dimensional embedding plot using Uniform Manifold Approximation and Projection ( https://arxiv.org/abs/1802.03426 ), as shown in Additional file 1 (Figure 19-22). The results suggest that mapping query cells onto the space derived from database cells lessens the batch effects. These results have been added to the revised manuscript and the additional figures so that the reader can easily see the effects of the projection.

Please note that our manuscript is not the first describing the effects of query projection in scRNA-seq. Li et al. clearly state that this technique is effective to reduce batch effects in the discussion of their RCA paper: “We therefore developed a novel algorithm named reference component analysis (RCA) that projects single-cell transcriptomes into a space defined by variability in a reference dataset. RCA clustered the benchmark data with high accuracy, indicating that reference-guided clustering can greatly reduce technical variation and batch effects.”

In addition, the nearest-neighbor search may be used as subroutine to correct batch effects. For example, Haghverdi et al. ( https://doi.org/10.1038/nbt.4091 ) have recently proposed a method identifying the mutually nearest neighboring cells across batches before correcting batch effects. This method assumes that the batch-effect variation is smaller than the biological variation between different cell types, and the authors verified the effectiveness of their approach under this assumption using several scRNA-seq datasets. Therefore, we predict that our cell search method is also usable even with non-negligible batch effects, at least for the purpose of cell typing.

In the previous manuscript, we separately referred to the RCA method by Li et al. and the canonical correlation-based method by Butler et al. in two distant sections. We added a reference to Butler et al. in the discussion so that the reader can easily find this mentioned in the context of batch effect removal.

*3) How do the authors measure the significance of the search outcome? If I search a random expression profile, will I still retrieve matches? This must be addressed carefully and convincingly.*

We have implemented an additional function to address this important point. CF may retrieve far distant cells because it always detects all the nearest neighboring cells of a query cell.

Therefore, even if expression profiles are completely randomized, it returns all the neighbors specified by the parameter *k* . In fact, the user can quantitatively estimate the similarities between the query cell and its neighbors by the Hamming distances of their hash values.

However, this method is neither intuitive nor explicitly described in our previous manuscript. To address the concern of reviewer #1, we added a function to calculate the estimator of the cosine similarity between a query and its neighboring cell. The signature of the function is “CellFishing.similarities(neighbors, database),” where “neighbors” and “database” denote a result object of cell search and a database object, respectively, and returns a similarity matrix (rows: neighbors, columns: queries) of floating-point numbers between -1 and 1. The estimation method is described within “Similarity estimation” in the methods.

We conducted a simulation study mimicking the situation in which a cell type corresponding to query cells does not exist in the database. As we have described in the manuscript, the cosine similarity is an important criterion to quantitatively measure the significance of the search outcome. The details are described in “Evaluation of the search outcome and DEG detection” of the results.

*4) I am not a fan of comparisons. Every landmark paper should present sufficient novel perspectives. For example, can the author show some use cases such as rare cell discovery?*

To address this understandable criticism, in the revised manuscript, we have added a novel function to detect query cell DEGs compared to their nearest neighbors at the single-cell resolution. We believe that this is an important novelty because no other cell search software provides this kind of functionality; it can be used to estimate the state of the query cells, such as by inferring their developmental stages and distinguishing cells in abnormal versus normal states. The method is rapid and simple, thus the user can quickly enumerate plausible DEGs and interpret the results. A drawback of this new function is that it requires more disk space to store raw UMI counts. We curb this increase by compressing the count matrix of filtered genes and decompressing the required segments on request. As we have detailed in the manuscript, this method successfully detected subtle differences between immature B cells and other subtypes of B lymphocytes, with an increase of a file size of approximately 3-5 times.

Scalability is also an important novelty of CellFishing.jl. To the best of our knowledge, CellFishing.jl is the first single-cell search method scalable to more than one million cells with high accuracy and throughput. The search time is sublinear to the database size due to the indexing technique, as described in “Scalability for large data sets” of the results. We predict that our method is applicable practically to even much larger datasets containing tens of millions of cells.

Response to reviewer #2

*Page 4: "All mouse cells (1,886 total) of the Baron2016 data set were excluded …." Do the authors mean that all human cells were excluded, since the other three datasets are all mouse data?*

No, all mouse cells are excluded. This is because the majority of the cells of Baron2016 (10,455 cells) are derived from human (human, 8,569 cells and mouse, 1,886 cells). Here, our focus is to benchmark the runtime performance of the randomized singular value decomposition (SVD), and thus we avoided merging cells from different species in order to simplify the task. Also, our data sets contain planarian cells (Plass2018); thus, “the other three datasets are all mouse data” is not correct.

*Page 4: "It must be noted that in our application, obtaining exact singular vectors is not particularly important;" How did the author select the number of top singular values in practice?*

*What if the number of singular vectors needed to capture the high variability subspace corresponds to a relative high error, such as rank > 45 in Figure 2b?*

We appreciate the reviewer for raising such an important point. The observation from our preliminary experiments is that the search quality is fairly robust to the selection of this parameter (e.g., setting the parameter D between 30 and 70), and D = 50 is a reasonable default in cases where the database contains a wide variety of cell types. This is the reasoning behind why we used D = 50 for all the experiments in our manuscript without tuning for each data set. Some researchers may prefer to perform the JackStraw test (Plass et al. 2018) or use the Marchenko-Pastur distribution (Macosko et al. 2015, Shekhar et al. 2016) to find the number of statistically significant principal components from the data matrix. It is possible to automate these testing procedures in CellFishing.jl; however, we expect that this will not have critical effects in terms of cell-typing accuracy.

The relatively high error in a high-rank region is mainly due to the biological signals being too weak to distinguish them from random noise. In the first step of randomized SVD, it attempts to find the range of a data matrix caused by signals, and therefore noise and inconspicuous signals are ignored. If the data has measurable biological signals, we expect that randomized SVD captures them even when they are present in a high-rank region. This is consistent with the results from Shekhar2016 and TabulaMuris. In Shekhar2016, the relative error becomes larger (approximately 0.1) in the low-rank region (rank, approximately 20), whereas in TabulaMuris, the relative error remains low (approximately 0.05) even in the high-rank region (rank, approximately 50). This can likely be explained due to TabulaMuris having more biological variability because it encompasses a more wide variety of cell types than Shekhar2016 does.

*Page 5: "As a result, we limited the length of the bit vectors to 128 or 256 bits in the following experiments." The example data used by the author have cell numbers on a very similar scale, so it is not a problem to use 128 or 256 bits after investigating Figure 3b. However, in real practice, such investigation is not possible. How do the authors set T in their package? Is there a way to select automatically T based on the cell number?*

The parameter T controls the tradeoff between search speed and accuracy. In our experiments, we found that setting T = 128 is sufficient to distinguish different cell types, and thus, this value was used as the default for T. This is the case even if the database size changes; the variance of the similarity estimator depends on T but not on the size of the database. We think that using much longer bits (e.g., T = 1024) will not crucially contribute to more precise discrimination of cells since scRNA-seq data itself is noisy.

*Page 6: In self-mapping evaluation, it would be informative to also show CellFishing's*

*performance compared with single-cell clustering methods.*

We assume that reviewer #2 expects us to evaluate the performance of unsupervised clustering methods developed for scRNA-seq data and compare it with that of our supervised search method. Here, we evaluated single-cell consensus clustering (SC3) developed by Kiselev et al. in 2017 ( https://doi.org/10.1038/nmeth.4236 ) as the standard clustering method because it is one of the most highly cited papers in this field since its publication last year. Because the performance of an unsupervised clustering method cannot be evaluated with the consistency score or Cohen’s kappa score, we used the adjusted Rand score, as can be seen in the original SC3 paper. We performed clustering using SC3’s hybrid approach (i.e., unsupervised clustering with 5,000 random cells and cluster prediction by a support vector machine; see Kiselev et al. for details) with the default parameters for each of the four datasets used in our manuscript. The median of the adjusted Rand scores of the five trials were 0.33 (Baron2016-human), 0.49 (Shekhar2016), 0.09 (Plass2018), and 0.37 (TabulaMuris-chromium). We also evaluated the adjusted Rand scores of CellFishing in the same manner as the other scores. The median of the adjusted Rand scores of the five trials were 0.97 (Baron2016-human), 0.98 (Shekhar2016), 0.51 (Plass2018), and 0.96 (TabulaMuris-chromium). Please note that this is not a completely fair comparison, as CellFishing is a supervised method using the label of the nearest neighboring cell of a query, while SC3 is not; a more fair comparison would be possible if there were semi-supervised clustering methods for scRNA-seq. The reproducible scripts and analysis results are included in the additional files.

*Page 9: Figure 8 is difficult to read. To better demonstrate the results on mapping across different protocols, I'd suggest the authors to also plot a heatmap containing only overlapping cell type labels from the two platforms.* We added a new heatmap plot of overlapping cell types to Additional file 1 (Figure 48, 50).

However, we did not use this plot in the primary manuscript because it loses some information in exchange for brevity.

*Page 13: "Also, the computation of FTT is significantly faster than that of log transformation."*

*Regardless of the computation time, how should the users select from the log or FTT transformation? How to check if the Poisson noise assumption is reasonable?*

The idea of using FTT originated from Wagner et al. 2018, wherein they reported that FTT is better than log transformation in terms of stabilizing the mean-variance relationships of genes using an inDrop dataset (refer to their paper, Figure 3). This observation is compelling; thus, we added FTT as an option of transformations. We predict that the user can perform the same procedure as Wagner et al. in order to judge which transformation is more suitable for their data.

However, this requires a dataset without biological variability in the same experimental protocol, and thus is not always possible to perform. Also, using FTT in scRNA-seq is not widely accepted by the community because it is a relatively new idea. In our experiments, we could find no evidence suggesting that FTT is reasonably preferable over log transformation in terms of cell-typing accuracy. Therefore, in CellFishing.jl, we use log transformation as the default variance-stabilizing transformation instead of FTT.

*Page 14: "(3) batch effects between the query and the database are mitigated." I don't think dimension reduction can mitigate the batch effects. If there exist batch effects, some top components in the reduced space may actually represent them.*

We have meticulously addressed this concern as the second response to reviewer #1 as well as within the manuscript. We will not repeat the details here; however, in summary, projecting query cells onto the space spanned by the variability of database cells mitigates batch effects because the space does not embrace unwanted variability between the query and the database cells.

*Page 15: Is SRP-LSH proposed by the authors? There are no references for this method in the manuscript.*

No, to the best of our knowledge the basic idea of SRP-LSH dates back to the work of Goemans and Williamson ( https://doi.org/10.1145/227683.227684 ) in 1995; however, its application in similarity estimation was first proposed by Charikar in 2002 ( https://doi.org/10.1145/509907.509965 , cited in the manuscript). We added a reference to Charikar’s paper just after the first mention of SRP-LSH.

*Page 16: In SRP-LSH, how does the dimension of the hyperplanes affect the estimation accuracy of the similarity function?*

The dimension of the hyperplanes is always D minus one because the random hyperplanes are drawn in the D-dimensional space, where D refers to the dimension of the reduced space computed by SVD. Therefore, the discussion concerning the dimension of the hyperplanes is essentially the same as described in the selection of D above.
